# A New Opportunity for “Old” Molecules: Targeting PARP1 Activity through a Non-Enzymatic Mechanism

**DOI:** 10.3390/ijms24108849

**Published:** 2023-05-16

**Authors:** Pablo Iglesias, Marcos Seoane, Irene Golán-Cancela, Máximo Fraga, Victor M. Arce, Jose A. Costoya

**Affiliations:** 1 Molecular Oncology Laboratory MOL, Departamento de Fisioloxía, Centro Singular de Investigación en Medicina Molecular e Enfermidades Crónicas (CiMUS), Facultade de Medicina, Universidade de Santiago de Compostela and Instituto de Investigación Sanitaria de Santiago de Compostela (IDIS), 15782 Santiago de Compostela, Spainvictor.arce@usc.es (V.M.A.); 2 Departamento de Anatomía Patolóxica e Ciencias Forenses, Universidade de Santiago de Compostela and Instituto de Investigación Sanitaria de Santiago de Compostela (IDIS), 15782 Santiago de Compostela, Spain

**Keywords:** poly(ADP-ribose) polymerase-1, PARP inhibitors, neoplasm, cancer, animal disease models

## Abstract

In recent years, new therapies have been developed based on molecules that target molecular mechanisms involved in both the initiation and maintenance of the oncogenic process. Among these molecules are the poly(ADP-ribose) polymerase 1 (PARP1) inhibitors. PARP1 has emerged as a target with great therapeutic potential for some tumor types, drawing attention to this enzyme and resulting in many small molecule inhibitors of its enzymatic activity. Therefore, many PARP inhibitors are currently in clinical trials for the treatment of homologous recombination (HR)-deficient tumors, BRCA-related cancers, taking advantage of synthetic lethality. In addition, several novel cellular functions unrelated to its role in DNA repair have been described, including post-translational modification of transcription factors, or acting through protein–protein interactions as a co-activator or co-repressor of transcription. Previously, we reported that this enzyme may play a key role as a transcriptional co-activator of an important component of cell cycle regulation, the transcription factor E2F1. Here, we show that PARP inhibitors, which interfere with its activity in cell cycle regulation, perform this without affecting its enzymatic function.

## 1. Introduction

As the cell cycle progresses, the cell divides and this division involves the duplication of its genetic material. Therefore, the cell cycle is a highly regulated process that integrates many signals from the membrane and from cellular pathways involved in controlling genetic integrity to ensure that genetic damage does not occur. The E2F/RB pathway is key to this regulation, and indeed mutations affecting this pathway induce aberrant cell cycle activity and are highly associated with many types of cancer [1]. As a result, activation of cyclin/CDKs complexes phosphorylates pRB family members, reducing their ability to interact with target proteins, and thus altering their biological functions [2]. 

Consistent with this, several reports have shown that the E2F/RB pathway is regulated by independent cell cycle regulators, including members of the poly(ADP-ribose) polymerase gene family [3,4]. Of the 17 identified members of the poly(ADP-ribose) polymerase (PARP) family, most are enzymes capable of attaching poly(ADP-ribose) units to proteins or DNA using NAD^+^ as a substrate, but only PARP1, -2, and -3 share a significant identity in their catalytic domains [5].

Given the importance of the catalytic activity of PARP1 in the process of genome repair, the development of molecules capable of inhibiting this activity becomes a desirable pharmacological target, primarily for their use as radiosensitizers or enhancers of other agents capable of inducing DNA damage [6,7]. However, the application of these inhibitors is not limited to their chemopotentiating effect, but they have also been tested as monotherapies in tumors with inactivated BRCA1 and/or BRCA2, as in the case of some breast and ovarian adenocarcinomas. These severe defects in homologous recombination repair make these types of tumors highly susceptible to treatment with agents capable of disrupting or collapsing replication forks compared to cells carrying functional copies of BRCAs [8,9]. This paved the way for clinical trials in which PARP1 inhibitors showed success when given alone or in combination with other chemotherapeutic agents, with some cases, such as olaparib, rucaparib, and niraparib leading to regulatory approval [10,11,12,13].

In addition to its involvement in DNA repair, PARP1 has been identified as having several important functions in other biological processes, including cell division and transcriptional regulation. [14]. Among these, PARP1 is known to alter gene expression by acting as a co-activator or a co-repressor of transcription in a promoter-specific manner, independent of its catalytic activity. Indeed, PARP1 can upregulate the transcriptional activity of E2F1 through its function as a co-activator during the re-entry of quiescent cells into S phase [3,4]. Recently, we have shown how PARP1 regulates the transcriptional function of E2F1 through its interaction at the promoters of E2F1 target genes and how PARP1 inactivation leads to a reduction in proliferation rate in a gliomagenesis model [15]. Here, we show how this transcriptional modulation can be affected by pharmacological treatment with PARP1-interacting molecules.

## 2. Results and Discussion

### 2.1. In Vitro Effect of PARP Inhibitors on the Interaction of PARP1 and E2F1 

The activity of the transcription factor E2F1, especially in the G1/S transition, is strictly dependent on the phosphorylation of pRB. Therefore, while a hypophosphorylated pRB is capable of binding and inhibiting E2F1, the hyperphosphorylation of this protein produces a series of allosteric changes that drastically modify their interaction, leaving the E2F1 factor free. This circumstance is used by many tumor types that, by inactivating pRB, deregulate the activity of E2F1, and thus the cell cycle to their own advantage [2]. 

**Figure 1:**
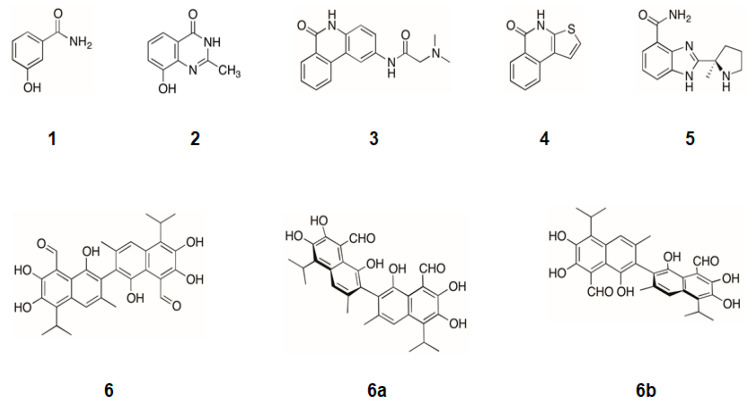
Structure of PARP1 inhibitors and gossypol, an inhibitor of PARP1 protein–protein interactions. Compound **1**, 3-aminobenzamide; compound **2**, NU1025; compound **3,** PJ34; compound **4**, TiQ-A; compound **5,** ABT-888; compound **6**, gossypol. Gossypol is obtained from its natural source (cotton) as a racemic mixture of two atropisomers where the (-)-gossypol isomer (**6a**) specifically interferes with PARP1 protein–protein interactions.

In a previous work [15], we have previously described that PARP1 acts as a co-activator of E2F1, and that its deficiency reduces the transcriptional activity of E2F1, thereby reducing tumor growth by decreasing E2F1 hyperactivation. Therefore, here we tested whether this decrease in E2F1 transcriptional activity could be achieved by using several representative examples of the different generations of chemical inhibitors of PARP1. Among the classical inhibitors, 3-aminobenzamide (Figure 1, compound **1**) stands out as one of the first nicotinamide analogues used for this purpose [16]. Within the second generation, we used derivatives of benzoquinolines and benzoquinolinones, such as TiQ-A and PJ34 (Figure 1, compounds **3** and **4**, respectively), and quinazolinones, such as NU1025 (Figure 1, compound **2**), one of the PARP1 inhibitors used in a pioneering synthetic lethality study in BRCA1/2 deficient tumors [9]. Similarly, we also include one of the third-generation inhibitors of PARP1, veliparib (ABT-888), that has demonstrated its efficacy in different clinical phase trials and in different tumor types [17]. Finally, we included gossypol (Figure 1, compound **6**), a compound of natural origin with the ability to specifically block protein–protein interactions of PARP1 mediated by the central BRCT (BRCA1 C-Terminus) domain, as in the case of PARP1 and E2F1 [18].

**Figure 2:**
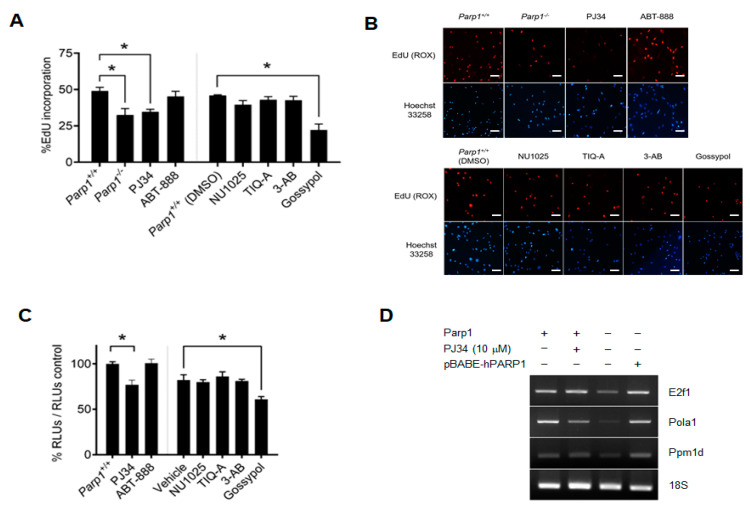
Effect of PARP1 inhibition on E2F1 transcriptional activity. (**A**), EdU incorporation assay in MEF treated with the PARP1 inhibitors PJ34 (10 µM), ABT-888 (10 µM), NU1025 (100 µM), TIQ-A (50 µM), 3-AB (5 mM), and gossypol (25 µM). A vertical line separates cells treated with inhibitors dissolved in water and inhibitors dissolved in DMSO (* = *p* < 0.01). (**B**), representative images of results presented in panel A. Cell nuclei were counterstained with Hoechst 33258 (bisbenzimide). Scale (20×) = 25 μm. (**C**), luciferase activity assay in HEK293 transfected with E2F-Luc plasmid treated with PARP1 inhibitors. A vertical line separates cells treated with inhibitors dissolved in water and inhibitors dissolved in DMSO. Cells were synchronized prior to treatment, and results were normalized to its corresponding vehicle (water or DMSO). Shown is a representative experiment of three independent replicates (* = *p* < 0.01) (**D**), semiquantitative RT-PCR of E2F1 transcriptional targets. RNA was isolated from *Parp1^+/+^*, *Parp1^−/−^*, and *Parp1^−/−^* transduced with pBABE-hPARP1 and specific PCR products from each gene indicated in the figure were run on an agarose gel. Results are representative of three independent experimental replicates.

To evaluate the effect the different PARP1 inhibitors on cell proliferation, we used mouse embryonic fibroblasts (MEFs) obtained from either *Parp1^+/+^* or *Parp1^−/−^* mice. Cells were seeded and serum-starved to maintain them in the G1/S transition, and then treated with the inhibitors. Cell proliferation was significantly decreased in *Parp1^−/−^*, as compared with *Parp1^+/+^* and cells (Figure 2A), which is consistent with the role of PARP1 as a co-activator of E2F1 in the initiation of S phase. Interestingly, cell proliferation was also reduced after treatment of *Parp1^+/+^* cells with PARP1 inhibitors, more effectively in the case of cells treated with PJ34 or with gossypol (Figure 2B). Considered together, these data indicate that PARP1 inhibition significantly reduces E2F1 transcriptional activity and cell proliferation, consistent with the hypothesized contribution of PARP1 as a co-activator of this transcription factor. Chemical inhibition of the enzymatic activity of PARP1 has an effect similar to that observed in cells lacking this protein, whereas specific inhibition of the protein–protein interaction, as in the case of gossypol treatment, appears to have an even greater effect. 

To further demonstrate the involvement of E2F transcriptional activity, we performed a luciferase activity assay using HEK293 cells transfected with the E2F-Luc vector and synchronized by the double thymidine method. Once released from arrest, cells were treated with the different inhibitors, as shown in Figure 2C. The enzymatic inhibition of PARP1 reduced the transcriptional activity of E2F1 in a manner similar to the PARP1 deficiency that we previously reported [15]. Within the experimental groups, we found that PJ34 is possibly the molecule, among the classical inhibitors of PARP1, that reduces this transactivation to a greater extent, while the luciferase activity is reduced by almost 40% compared with the untreated control in cells treated with gossypol. 

To verify whether this effect on the transcriptional and proliferative activity correlates with the subcellular localization of the proteins, we co-transfected vectors containing the EGFP-PARP1 and RFP-E2F1 fusion proteins in HEK293 cells. These cells were maintained at the G1/S transition until treated with the inhibitors PJ34 and gossypol. Supplementary Figure S1A shows the co-localization of E2F1 and PARP1 in control cells, while treatment with the PJ34 inhibitor alters their subcellular localization, particularly of E2F1. At 4 and 6 h, after treatment, the intensity of E2F1 decreases notably, suggesting that the stability of the transcription factor is somehow compromised (Supplementary Figure S1A). This is not the case for cells treated with gossypol (Supplementary Figure S1A), in which a slight delocalization that affects PARP1 to a greater extent is observed as soon as after 4 h of treatment. However, the possible side effects of gossypol treatment must be taken into consideration, since it is a mimetic of the BH3 domain of the anti-apoptotic protein Bcl-2. Therefore, gossypol can antagonize Bcl-2 function, which can lead to cell apoptosis [19,20]. Considered together, these data indicate an effect of PJ34 and, to a lesser extent gossypol, on the interaction between PARP1 and E2F1. This effect is consistent with the functional assays, in which these treatments reduce the proliferation and transcriptional transactivation of treated cells. 

Since the effects of PJ34 and gossypol treatments were observed in cells released from synchronization at the G1/S transition, we speculated that changes in the stability of the transcription factor E2F1 might be involved. Therefore, we treated cells expressing the fusion proteins with cycloheximide, which inhibits protein synthesis by blocking mRNA translation, and monitored protein stability. As can be seen in Supplementary Figure S1B, E2F1 levels vary very slightly in both control and gossypol-treated cells. In contrast, a pronounced variation is observed in cells treated with PJ34 after 6 h of treatment, and thus suggest a possible effect of this molecule on the stability of E2F1.

To study the effect that the presence or absence of PARP1 could exert on the activity of E2F1, we also analyzed the mRNA levels of several genes regulated by E2F1 in fibroblasts, using semiquantitative PCR (RT-PCR). Our results show that the presence of PARP1 significantly reduces the transcriptional activity of the E2F1 protein, as well as other transcriptional targets of this factor, such as DNA polymerase α (Pola), whose induction occurs in early S phase, or the phosphatase Wip1 (Ppm1d). Indeed, the fact that we can rescue this effect by re-expressing a copy of PARP1 in *Parp1^−/−^* fibroblasts clearly demonstrates the role of PARP1 as a co-activator of the transcriptional activity of E2F1 (Figure 2D).

### 2.2. Chemical Inhibition of PARP1/E2F1 Interaction in a Model of Oncogenesis

As a final step in our study on the PARP1/E2F1 interaction, we investigated its possible consequences on oncogenesis. To this end, we took advantage of our model of gliomagenesis, based on the inactivation of a tumor suppressor (retinoblastoma) and the gain of function of an oncogene (HRAS^V12^) [19]. Since inactivation of retinoblastoma causes numerous cell cycle aberrations, mainly due to the uncontrolled behavior of E2F1, this model is an excellent test bench to study the effects of PARP1 in the context of hyperactivation of the E2F1 factor.

**Figure 3:**
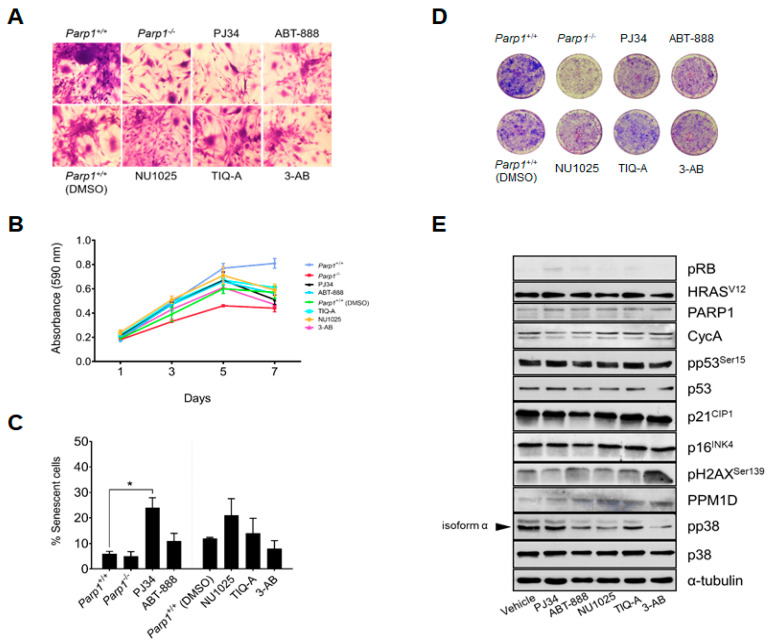
Effect of PARP1 inhibitors on primary astrocytes. Postnatal-day-3 astrocytes obtained from *Parp1^−/−^* c*Rb^−/−^* HRAS^V12^ or *Parp1^+/+^* c*Rb^−/−^* HRAS^V12^ mice were treated in vitro with PARP1 inhibitors. (**A**), morphological changes in cells stained with crystal violet. (**B**), proliferation rate. Cells were stained with crystal violet, and cell number was determined by spectrophotometry. (**C**), percent of senescent cells, obtained by quantification of SA-β-galactosidase activity. Shown is a representative experiment of three independent replicates (* = *p* < 0.001). (**D**), colony formation. Astrocytes were fixed with methanol-acetic acid (3;1, *v*/*v*) and stained with 0.1% (*w*/*v*) crystal violet in PBS on day 7 of culture. (**E**), biochemical analysis of the main DDR checkpoints and transcriptional targets of E2F1. All results are representative of, at least, three independent experiments.

As we have already shown [15], *Parp1^+/+^* c*Rb^−/−^* HRAS^V12^ astrocytes obtained from P3 neonates present great heterogeneity in their shape and size, together with several morphological alterations, such as the presence of abundant cytoplasmic extensions. Loss of contact inhibition and formation of growth foci in the culture plates, which reflect the existence of a transformed phenotype, was also observed in these cells. In contrast, *Parp1^−/−^* c*Rb^−/−^* HRAS^V12^ astrocytes, display a similar phenotype, but lower cell density and a significant decrease in the number of foci (Figure 3D). Altogether, these findings suggest that the degree of transformation is considerably lower in *Parp1^−/−^* c*Rb^−/−^* HRAS^V12^ astrocytes than in control cells. Interestingly, a similar effect, including a reduction in the number of foci was observed when *Parp1^+/+^* c*Rb^−/−^* HRAS^V12^ astrocytes were treated with the different PARP1 inhibitors (Figure 3A), especially in the case of PJ34, although these effects were lower than in PARP1-deficient astrocytes (Figure 3B).

To check whether this effect of PARP1 inhibitors on cell proliferation could be reflected in oncogene-induced senescence (OIS), we quantified the number of senescent cells in each experimental group using an SA-β-galactosidase activity assay (Figure 3C). Not surprisingly, the highest proportion of senescent cells, was observed in cells treated with PJ34, and to a lesser extent NU1025, which would explain their reduced proliferation compared to the rest of the experimental groups. Interestingly, we did not observe an increase in senescence in *Parp1^−/−^* astrocytes, which could be explained by an adaptive phenomenon induced by the chronic absence of PARP1. In contrast, no significant increase in apoptosis levels was observed in any experimental group (Supplementary Figure S1C).

In scenarios where there is DNA hyper-replication, for example, due to the activation of an oncogene, the response to DNA damage is activated, leading to the appearance of oncogene-induced senescence (OIS). This mechanism of cellular defense depends on several checkpoints, such as p16^INK4a^, p21^CIP1^ and, most importantly, p53. This tumor suppressor constantly monitors the cellular stress and, if necessary, induces tumor suppressor signaling pathways, resulting in cell arrest or cell apoptosis [21,22,23,24,25]. With this in mind, we wanted to observe whether the morphological changes observed previously would be reflected in the proteins involved in the DNA damage checkpoints, and for this purpose, we carried out an exhaustive biochemical analysis, which we present below in Figure 3E.

Regarding the status of the DNA damage response (DDR) checkpoints, a significant increase in activated p53 protein (p-p53^S15^) was observed in virtually all groups treated with PARP1 inhibitors. This can be largely attributed to the inhibition of PARP enzymatic activity, which would potentiate the damage caused by the HRAS^V12^-induced DNA hyper replication. Interestingly, this damage-enhancing effect was not observed in astrocytes treated with ABT-888 and 3-AB (as reflected by phospho-histone H2AX levels), while phospho-histone H2AX levels were significantly reduced in the case of PJ34.

On the other hand, if we observe the levels of the p21^CIP1^ cycle inhibitor, we observe that these also increase in almost all the treated groups, which would explain the lower proliferation of these cells with respect to the untreated control. Regarding the transcriptional activity of E2F1, we observed two very important facts: First, the levels of cyclin A, a bona fide transcriptional target of E2F1, were reduced in c*Rb*^−/−^ HRAS^V12^ astrocytes treated with PJ34. Second, the levels of phosphorylated p38 were higher in cells treated with PJ34, which may be related to the slight decrease in Wip1 phosphatase (Ppm1d), observed in PARP1-deficient astrocytes and in those treated with vehicle and PJ34. This finding may explain the higher level of senescence in PJ34-treated astrocytes, since downregulation of Wip1 on c*Rb^−/−^* HRAS^V12^ astrocytes sensitizes them to oncogene-induced senescence, as we previously reported [26].

In summary, our data demonstrate that chemical inhibition of PARP1, would increase DNA damage by blocking the enzymatic activity of PARP, involved in several mechanisms of DNA repair induced by replication stress. This increase in DNA damage, evidenced by the presence of high levels of phosphorylated p53, would increase the levels of p21 in the groups treated with inhibitors, and thus increase the percentage of senescent cells. In addition, in cells treated with PJ34, there is a lower transcriptional activity of E2F1, which is hyperactivated due to the deletion of *Rb*. This reduced activity of E2F1 translates into lower levels of the proteins regulated by this transcription factor: E2F1 itself, cyclin A, and Wip1 [27]. Dephosphorylation of p38MAPK by Wip1 greatly reduces its activity, making astrocytes more susceptible to transformation. On the other hand, inactivation of Wip1 and consequent re-activation of p38MAPK restores the ability of the cells to enter senescence and reduce their proliferation rate [26,28].

### 2.3. Rescue from PARP1 Deficiency

**Figure 4:**
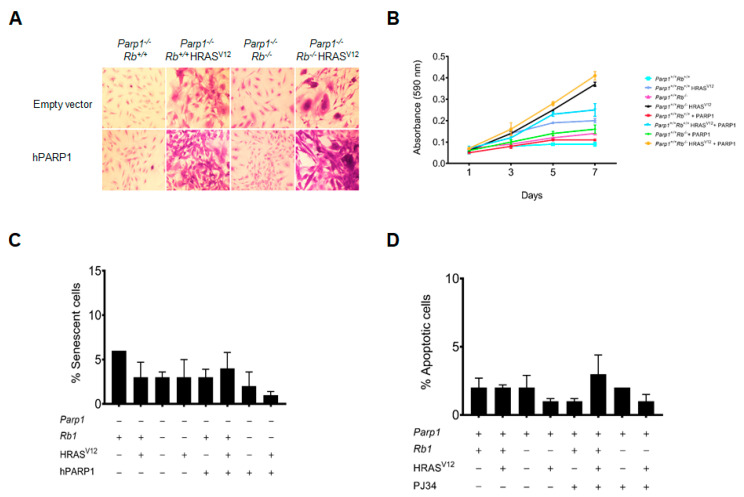
Effect of partial restoration of PARP1 catalytic activity. Primary astrocytes obtained from *Parp1^−/−^* c*Rb^flox/flox^*, *Parp1^−/−^* c*Rb^flox/flox^* HRAS^V12^, *Parp1^−/−^* c*Rb^−/−^*, or *Parp1^−/−^* c*Rb^−/−^* HRAS^V12^ mice were transduced with a copy of hPARP1 with reduced catalytic activity. (**A**), morphological changes in cells stained with crystal violet. (**B**), proliferation rate. Cells were stained with crystal violet, and cell number was determined by spectrophotometry. (**C**), percent of senescent cells, obtained by quantification of SA-β-galactosidase activity. (**D**), percent of apoptotic cells. Apoptotic nuclei were stained with Hoechst 33258. All results are representative of, at least, three independent experiments.

As a further demonstration of our previous experiments, we investigated the effects of PARP1 inhibition by PJ34 treatment on the proliferation of PARP1-deficient cells, in which a copy of hPARP1 was introduced by retroviral transduction. Supplementary Figure S2B–D, shows the effect of PARP1 inhibition with PJ34 on the proliferation and senescence in the different genotypes investigated in this experimental setting. As can be observed in Figure 4, the rescue of the PARP1-deficient phenotype, by re-expression of a copy of PARP1, has a small but appreciable effect on the proliferation in all cells expressing oncogenic Ras as well as in those deficient in pRB. The morphology of the knock-out astrocytes with re-expressed PARP1 should also be highlighted, since they show a more pronounced degree of transformation and, therefore, more similar to *Parp1^+/+^* astrocytes than to the control *Parp1^−/−^* (Figure 4A,B). On the contrary, while PARP1 re-expression does not appear to have a significant effect on astrocytes in terms of senescence, the level of apoptosis in Ras astrocytes is significantly reduced when a copy of PARP1 is present (Figure 4C,D). In this regard, we should also mention that in an a posteriori analysis the wild-type copy of PARP1 that we used in the present work incorporated a SNP, resulting in the substitution of alanine 762 by a valine residue. This mutation leads to an increase in Km for trans-poly(ADP-ribosyl)ation and results in a decrease in enzyme activity by approximately 50% [29]. However, this finding further supports the hypothesis that the reduction in proliferation observed in PARP1-deficient astrocytes is due to protein–protein interaction with E2F1. 

### 2.4. PARP Inhibitors Reduce Tumor Number and Size In Vivo

**Figure 5:**
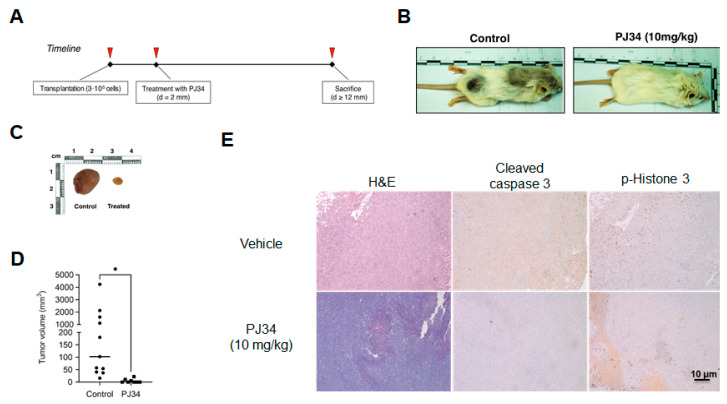
Effect of PARP1 inhibition in vivo. (**A**), timeline of the in vivo transformation assay. (**B**,**C**), effect of PJ34 on tumoral growth. *Parp1^+/+^* c*Rb^−/−^* HRAS^V12^ mice were injected subcutaneously with 3·10^6^ astrocytes and treated with vehicle (control) or PJ34 (10 mg/kg). (**D**), tumor volume in control (*n* = 11) or PJ34-treated (*n* = 8) mice. Representative images or results presented in panel B and **C** (* = *p* < 0.05). (**E**), caspase 3 and p-histone 3 expression in tumors obtained from control or PJ34-treated.

Finally, and to determine whether the transient inhibition of PARP1 could affect the transformation of modified astrocytes in vivo, we subcutaneously injected *Parp1^+/+^* c*Rb^−/−^* HRAS^V12^ astrocytes into the flank of the hindquarters of immunodeficient SCID mice (see Figure 5A for further details). As previously reported by our group [15], 100% of injected mice developed tumors. However, a single intraperitoneal treatment with PJ34 (10 mg/kg) significantly reduced tumoral masses as compared to those injected with vehicle (Figure 5B–D). Histological analysis of the specimens (Figure 5E) also revealed a marked decrease in phosphorylated histone H3 labeling in the tumors obtained from treated mice. 

## 3. Materials and Methods

### 3.1. Primary Cell Cultures

Cells were maintained in Dulbecco’s modified Eagle medium (Sigma-Aldrich, St. Louis, MO, USA) with 10% fetal bovine serum and 1% L-glutamine (GIBCO-Invitrogen, Waltham, MA, USA). Primary astrocytes were generated from both *Parp1*^+/+^ *cRb*^loxP/loxP^ and *Parp1*^−/−^ c*Rb*^loxP/loxP^ neonatal mice at P3. Oncogenic HRAS expression and deletion of *Rb* was also achieved by retroviral transduction using Phoenix-Eco cells [30] transfected with pBABE, pBABE-HRAS^V12^, PIG-puro, and PIG-CRE retroviral plasmids (a gift from P.P. Pandolfi). Transduced cells were selected by adding puromycin to the culture medium at 2 μg/mL.

### 3.2. PARP1 Inhibition and Other Pharmacological Treatments

The inhibitors 3-aminobenzamide (3-AB, sc-3501, Santa Cruz Biotechnology, Inc., Dallas, TX, USA), NU 1025 (sc-203166, Santa Cruz Biotechnology, Inc., Dallas, TX, USA), ABT-888 (sc-202901, Santa Cruz Biotechnology, Inc., Dallas, TX, USA), TIQ-A (sc-204916, Santa Cruz Biotechnology, Inc., Dallas, TX, USA), and PJ34 (528150, Sigma-Aldrich, St. Louis, MO, USA) were used to inhibit the catalytic activity of PARP1. In addition, an allosteric inhibitor of BRCT-mediated protein–protein interactions of PARP1, gossypol (G8761, Sigma-Aldrich, St. Louis, MO, USA), was included in some of the experiments.

For the E2F1 half-life assay, HEK293 cells were seeded in a 12-well multiwell plate at a density of 5·10^4^ cells per cm^2^ and transfected with equimolar amounts of the pEGFP-PARP1 (a gift from A. Chiarugi) and pRFP-E2F1 (a gift from B. Su). After synchronization with double-thymidine treatment, cells were released with fresh DMEM medium along with the treatments (PJ34 and gossypol) and their respective controls plus cycloheximide (C7698, Sigma-Aldrich, St. Louis, MO, USA) at a final concentration of 35 µM.

### 3.3. Co-Localization Studies

For co-localization studies, 5·10^4^ per cm^2^ HEK293 cells were seeded on EZ-multiwell slides (Millipore, Burlington, MA, USA). Cells were transfected using equimolar quantities of pEGFP-PARP1 (a gift from A. Chiarugi) and pRFP-E2F1 (a gift from B. Su) and synchronized by double thymidine treatment. Upon release from cell cycle block, cells were fixed with 2% paraformaldehyde (pH 7.4), nuclei were counterstained using DAPI (Invitrogen, Waltham, MA, USA), and fluorescent images from three independent experiments were obtained using a Leica TCS SP2 microscope.

### 3.4. Incorporation of 5-Ethynyl-2′-Deoxyuridine (EdU)

Mouse fibroblasts were seeded at 5·10^3^ cells per cm^2^ in 24-well plates. Culture medium was removed 24 h later and replaced with low-serum medium (0.5% FCS). After 48 h, the starvation medium was replaced with high-serum medium (15% FCS) along with PARP1 inhibitors for 16 h and subsequently treated with 10 μM 5-ethynyl-2′-deoxyuridine (EdU, Sigma-Aldrich, St. Louis, MO, USA) for two additional h. Nuclei were stained using Hoechst 3358 (Sigma-Aldrich, St. Louis, MO, USA) and fluorescence images were collected from three different experiments.

### 3.5. Luciferase Assays

MEF were seeded in 12-well plates at 2.5·10^3^ cells per cm^2^. Cells were subsequently transfected with vectors pE2F-Luc (a gift from M. Collado) and pCMV-β-Gal (Clontech, Mountain View, CA, USA). Cell cultures were lysed 48 h after transfection with 1× Passive Lysis Buffer (Promega, Madison, WI, USA) for 20 min at room temperature. Aliquots of each cell lysate were mixed with 1× Luciferase Activity Buffer (25 mM glycylglycine pH 7.8, 15 mM phosphate buffer pH 7.8, 15 mM MgSO_4_, 4 mM EGTA, 2 mM ATP, 1 mM DTT) and 1 mM d-luciferin (L9504, Sigma–Aldrich, St. Louis, MO, USA). Luciferase activity was measured using a Lumat LB 9507 luminometer (Berthold Technologies, Baden Württemberg, Germany). β-Galactosidase activity assay was performed in Z buffer (0.06 M Na_2_HPO_4_·7H_2_O, 0.04 M NaH_2_PO_4_·H2O, 0.01 M KCl, 0.001 M MgSO_4_, 0.05 M 2-mercaptoethanol) and *o*-nitrophenyl-β-d-galactopyranoside (ONPG). Samples were incubated at 37 °C for 1 h and absorbances were measured with a BioMate 3 spectrophotometer (Thermo Fisher Scientific, Waltham, MA, USA) at 420 nm. The data from three independent experiments were normalized using beta-galactosidase activity.

### 3.6. Animal Studies

Xenografts were established in SCID (Severe Combined Immunodeficiency) mice aged 10 to 12 weeks. Cell implantation was carried out by subcutaneous injection in the hindquarters with 3·10^6^ transduced astrocytes resuspended in 100 µL PBS 1×. Treatment with PJ34 (Sigma-Aldrich, St. Louis, MO, USA) was initiated once the tumor reached a minimum diameter of 2 mm with a single-dose of 10 mg/kg, injected intraperitoneally. Control animals were injected PBS in a similar manner. All tumors included in the analysis reached a minimum diameter of 4 mm and mice were euthanized when they approached a maximum diameter of 12 mm. Tumors were considered as ellipsoid in shape and their volume was calculated using the equation volume = 0.5 × (length × width) [31]. The conditional mouse strain for *Rb1* [32], was obtained from the Mouse Models of Human Cancer Consortium (MMHHC) repository. *Parp1^−/−^* strain [33] was a gift from Prof. de Murcia. All animal procedures were approved and performed according to the guidelines set out by the Institutional Ethics Committee for Animal Experimentation.

### 3.7. Immunoblot

Analysis of protein levels was carried out by immunoblot analysis of whole cell lysates using polyclonal antibodies against PARP1 (H-300, Santa Cruz), cyclin A (C-19, Santa Cruz), p53 (CM5, Novocastra, Newcastle, UK), p-p53 ser15 (9284, Cell Signaling), p38 (C-20, Santa Cruz), p-p38 (Thr180/Tyr182) (sc-17852, Santa Cruz), p16 (M-156, Santa Cruz), p-H2AX Ser139 (07-164 Upstate), p21 (M-19, Santa Cruz), PPM1D/WIP1 (H-300, Santa Cruz), and E2F1 (C-20, Santa Cruz), as well as monoclonal antibodies against pan-Ras-V12 (Ab-1, Calbiochem, Sigma-Aldrich, St. Louis, MO, USA), cyclin D1 (DCS6, Cell Signaling Technology, Inc., Danvers, MA, USA), α-tubulin (T5168, Sigma-Aldrich, St. Louis, MO, USA), β-actin (MAB1501, Millipore, Burlington, MA, USA), and Rb (554136, BD).

### 3.8. Immunohistochemistry

For immunohistochemical analysis, anti-cleaved caspase 3 (monoclonal, Cell Signaling Technology, Inc., Danvers, MA, USA) and phospho-histone H3 (polyclonal, Cell Signaling Technology, Inc., Danvers, MA, USA) were used as primary antibodies. Immunohistochemical analysis was performed using a universal second antibody kit that uses a peroxidase-conjugated labelled dextran polymer (Envision Plus, Dako, Glostrup, Denmark). The following primary antibodies were used: Anti-cleaved caspase 3 (monoclonal, Cell Signaling Technology, Inc., Danvers, MA, USA) and phospho-histone H3 (polyclonal, Cell Signaling Technology, Inc., Danvers, MA, USA).

### 3.9. RNA Isolation and Semiquantitative RT-PCR

Embryonic fibroblasts were seeded at a rate of 4·10^4^ cells per cm^2^ in 10 cm plates and synchronized for 44 h serodeprivation. Cells were released from arrest and left in factor-rich medium (DMEM 15% FBS) for 16 h before RNA extraction. RNA was isolated using TRIzol (Thermo Fisher Scientific, Waltham, MA, USA) and cDNA was obtained using SuperScript III First-Strand Synthesis System (Thermo Fisher Scientific, Waltham, MA, USA) [34]. Specific sequences of each gene were amplified using the following oligonucleotide pairs: qE2F1-F 5′ CTCGACTCCTCGCAGATCG 3′, qE2F1-R 5′ AGCTCGGCGAGAAAAGAAATC3′, qPOLA1-F 5′ GAAGAACGAGATCAGCAG 3′, qPOLA1-R 5′ CCACATAGCCTATCCCATCGTC 3′, qWIP1-F 5′ GATGTATGTAGCGCATGTAGGTG 3′, qWIP1-R 5′ GTTCTGGCTTGTGATCTTGTGT 3′, 18S-F 5′ TTGACGGAAGGGCACCACCAG 3′, 18S-R 5′ CTCCTTAATGTCACGCACGATTTC 3′.

### 3.10. Statistics

Statistical analysis was performed with ANOVA and Student’s *t*-test for multiple or simple comparisons, respectively. Tukey and Student–Neumel–Kaus tests were used for post-hoc analysis of ANOVA results. Mantel–Cox test was used to analyze Kaplan–Meier curves. In all cases, statistical significance was established at *p* < 0.05.

## 4. Conclusions and Future Perspectives

Functional inactivity of the tumor suppressor pRB present in various tumor types and transgenic animal models, leads to dysregulation of E2F1 transcriptional activity, which correlates with aberrant cell proliferation and, in some cases, with cell death [35,36,37]. The effects of pRB deficiency or hyperactivation of E2F1 have been extensively studied, both in relation to the initiation of the oncogenic process [2,38,39] and the induction of embryonic lethality in mice [40,41].

Based on studies by Simbulan-Rosenthal [3,4] and previous results from our group [9], we have shown that the PARP1 protein plays an important role in the transcriptional activity of E2F1 by modulating it at the G1/S transition. This modulation of E2F1 occurs through the direct interaction of both proteins, and in the case of PARP1, takes place through the central domain or self-modification, which contains a BRCT motif similar to other proteins that interact with E2F1. In studies published by our group and others [3,15], it has been demonstrated that the association of PARP1 and E2F1 occurs directly on the promoter of E2F1 and other transcriptional targets of E2F1, such as cyclin A. Similarly, we also observed that the hyperactivation of E2F1 transcriptional activity in pRB-deficient cells can be reduced if PARP1 is also absent, confirming the role of this protein as a co-activator of E2F1 [15]. 

Although this interaction does not seem to depend on the enzymatic activity of PARP1, since E2F1 is not poly(ADP-ribosyl)ated in vitro, we observed that treatment with an inhibitor reduces the expression of some E2F1 transcriptional targets involved in the transition between low- and high-grade gliomas, such as POLA1 polymerase or WIP1 phosphatase, in MEFs synchronized by serodeprivation [26,28]. Interestingly, a similar effect was observed when cells were treated PJ34, an inhibitor of PARP1 enzymatic activity, and with gossypol, a molecule capable of specifically blocking the protein–protein interactions mediated by the BRCT motif of PARP1 [18]. Gossypol is a naturally occurring compound usually obtained as a racemic mixture of two stereoisomers of which only the (-)-gossypol isomer has the ability to interfere with PARP1 protein–protein interactions. Mechanistically, this biological activity translates into the ability of this molecule to react to form a Schiff base by reacting its two aldehyde functional groups with the amino groups of lysines 438 and 441 of the BRCT domain, and thus blocking any possible interaction between PARP1 and other proteins. In the case of PJ34, this molecule lacks aldehyde groups that allow for a gossypol-like interaction with the BRCT domain of PARP1. It is well known that this inhibitor, which is developed by Inotek [42], similar to many other PARP1 inhibitors, considers nicotinamide (NAM) as a structural model to bind to its binding site in the enzyme’s catalytic center and block its activity. In addition to binding to the NAM pocket, this molecule can locate itself between the α-helix and the D-loop of tankyrase-1 (PARP-5a), both structural motifs that are also present in the domains of PARP1 catalytic agents, suggesting that, similar to PARP-5a, it is capable of binding two PJ34 molecules simultaneously at both binding sites [5].

In relation to these results, the assays with the EGFP-PARP1 and RFP-E2F1 fusion proteins not only confirm that the co-localization of PARP1 and E2F1 increases with time from the G1 to S phase, but in the case of cells treated with PJ34, the levels of the RFP-E2F1 fusion protein appear to decrease significantly. As seen below, in the same synchronized cells, whose protein translation had been inhibited by cycloheximide treatment, E2F1 levels decreased more sharply over time in the case of PJ34, suggesting that this inhibitor may affect the stability of E2F1.

Finally, we wanted to test whether this cooperation between both proteins also extends to an oncogenic context, which would allow us to modulate the activity of E2F1 by inhibiting PARP1. To perform this, we used a model of gliomagenesis already characterized by our group, which is largely based on the hyperactivation of E2F1 by conditional deletion of *Rb* [26]. As in non-transformed cells, PARP1 inhibition leads to reduced proliferation, in addition to the reduction in aggressiveness and cell transformation observed in control cells (*Parp1^+/+^* c*Rb^−/−^* HRAS^V12^), especially in the case of PJ34. However, we also observed that in the case of cells treated with PJ34, the percentage of senescent cells in the culture increased (Figure 3C), which would also explain the lower proliferation rate of astrocytes treated with this inhibitor, in addition to the generation of fewer foci or colonies compared to untreated control astrocytes (Figure 3D). Consistent with this, the biochemical analysis shows an increase in the levels of the cycle inhibitor p21CIP as well as of phosphorylated p53 in the case of the group treated with PJ34, which would explain the increase in senescent cells observed in this group. Regarding this result, it is important to consider that PJ34, similar to the rest of the inhibitors used, may have some off-target or non-specific effects that could confound the results obtained. A review of the available literature shows that PJ34, in addition to presenting a high affinity for the catalytic centers of PARP1 and PARP2, as expected since they are the ones with the greatest homology to each other, is also capable of binding to several members of its family (PARP3, -4, -5a, -5b, -14, -16) [5], as well as the metalloprotease MMP2 and the kinases PIM1 and PIM2, albeit with lower avidity [43]. Despite the apparent redundancy of the biological functions of the different PARPs, especially PARP1 and PARP2, more and more specific functions are being discovered for these proteins [44]. Therefore, it is logical to speculate that the combined inhibition of multiple members of this family may have more durable and/or profound effects than the deletion of PARP1 alone, and thus explain these differences between *Parp1^−/−^* c*Rb^−/−^* HRAS^V12^ and *Parp1^+/+^* c*Rb^−/−^* HRAS^V12^ treated with PJ34.

Moreover, noteworthy, although expected, is the decrease in Ppm1d (Wip1) and cyclin A levels in this group and in *Parp1^−/−^* c*Rb^−/−^* HRAS^V12^ cells, since both proteins are transcriptional targets of E2F1 itself. Interestingly, the levels of p38-α kinase (MAPK14) were also increased in the case of PJ34 treatment in contrast to control cells. As we have already observed in previous studies in our laboratory, the level of oncogenesis-induced senescence (OIS) in c*Rb*^−/−^ HRAS^V12^ astrocytes is closely related to the activity of the p38-α-specific phosphatase, Wip1. This phosphatase, once inactivated, allows p38 to activate and act as a brake on tumorigenesis by forcing astrocytes to slow down their proliferation and enter a senescent state [26,28]. In the same way, by reducing the transcriptional activity of E2F1, we also reduce the levels of Wip1, and therefore the proliferation of these cells that lack the protection of this phosphatase is affected by senescence mediated by oncogenes, and that in our case is HRAS^V12^.

To complement these results, we wanted to verify once again that the absence of PARP1 is responsible for the change in phenotype and aggressiveness of our astrocytes. In the results that we have presented previously, it can be clearly observed that the reintroduction of a copy of PARP1 with reduced catalytic activity increases the proliferation and the degree of transformation of *Parp1^−/−^* c*Rb^−/−^* HRAS^V12^ astrocytes, whereas, as in the previous experiment, the chemical inhibition of PARP1 by means of PJ34 has an antagonistic effect. We reached the same conclusion when injecting these astrocytes into SCID mice, since *Parp1^−/−^* c*Rb^−/−^* HRAS^V12^ cells had a very tumorigenic capacity compared to controls. In turn, a single-dose treatment (10 mg/kg) of the PJ34 inhibitor was sufficient to reduce the volume of the masses obtained in the treated mice. Finally, the immunohistological analysis of these tumor tissues confirmed that in the case of treatment with PJ34, proliferation is greatly reduced, as evidenced by the low labeling of phosphorylated histone H3 compared to control tissue. In the case of *Parp1*^−/−^ c*Rb*^−/−^ HRAS^V12^ astrocyte tumors, the labeling of proliferating cells was also reduced, but in contrast to what occurred in the in vitro oncogenesis studies, the levels of cell apoptosis were reduced inside the tumor.

In conclusion, in the present study, we have shown that treatment with the inhibitor PJ34 or the inhibitor of protein–protein interactions gossypol can reduce the transcriptional activity of E2F1 and the proliferation of the treated cells. Inhibition of PARP1 protects the cell against oncogenic stimuli by reducing its proliferative rate, both in vivo and in vitro, or by reactivating other cell signaling pathways involved in oncogenesis-induced senescence.

## Data Availability

The data that support the findings of this study are available from the corresponding author upon reasonable request.

## Supplementary Materials

The supporting information can be downloaded at: https://www.mdpi.com/article/10.3390/ijms24108849/s1.
