# Supplementary material for: A New Opportunity for “Old” Molecules: Targeting PARP1 Activity through a Non-Enzymatic Mechanism
Source: Int J Mol Sci. 2023 May 16;24(10):8849. doi: 10.3390/ijms24108849 (PMC10219095; doi:10.3390/ijms24108849)
Supplement: Supplementary file 1 [file ijms-24-08849-s001.zip › ijms-2378662-supplementary.pdf]

Supplementary Materials

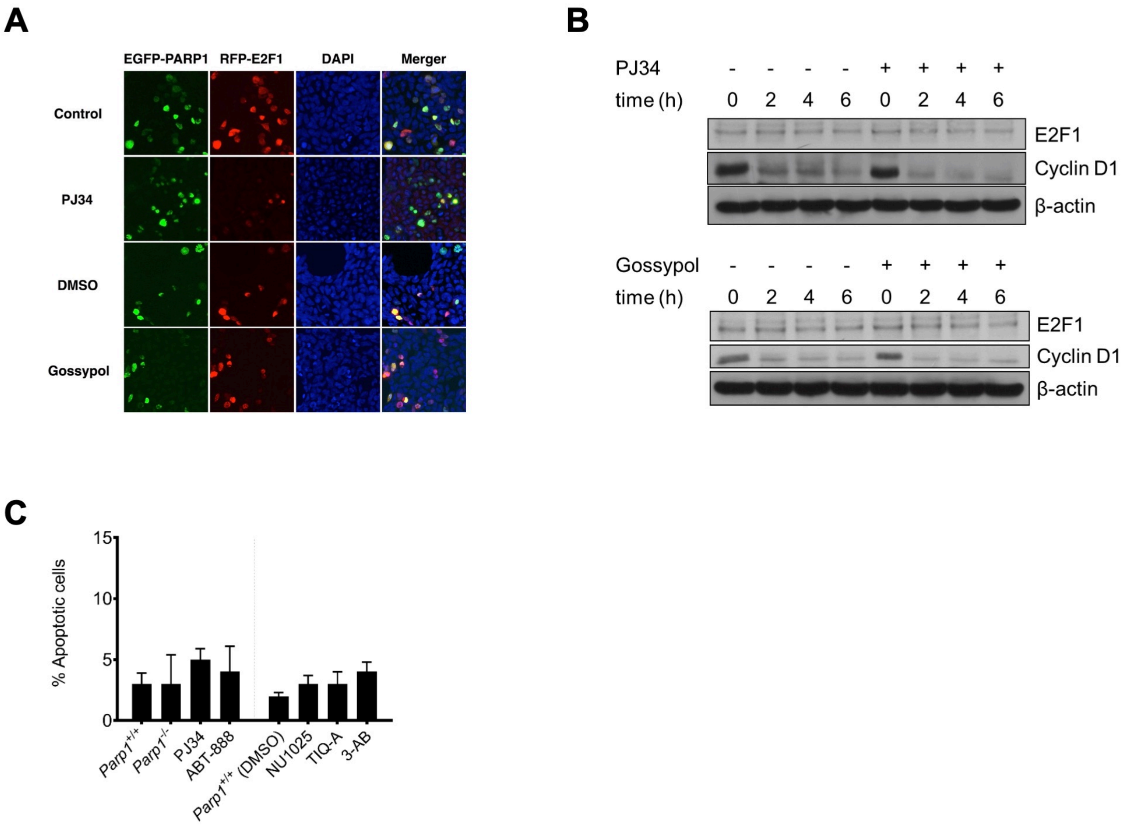

Supplementary Figure S1. A, co-localization of EGFP-PARP-1 and RFP-E2F-1 in HEK293 cells at 0, 2, 4 and 6 hours after arrest at the G1/S phase. Nuclei were counterstained with DAPI. The three overlapping fluorescence channels are shown at the right (63x). Results are representative of three independent experiments. B, Effect of cycloheximide treatment on E2F1 expression in control cells or in cells treated with PJ34 (10 µM) (upper) or gossypol (25 µM) (lower). Cyclin D levels are shown as a control for cycloheximide treatment, and beta-actin as a loading control. C, Effect of treatment with PARP1 inhibitors on cell apoptosis. Cells were treated with PJ34 (10 µM), ABT-888 (10 µM), NU1025 (100 µM), TIQ-A (50 µM), 3-AB (5 mM) and gossypol (25 µM), and apoptotic nuclei were detected by Hoechst 33258 staining. Each experiment was repeated at least three times.

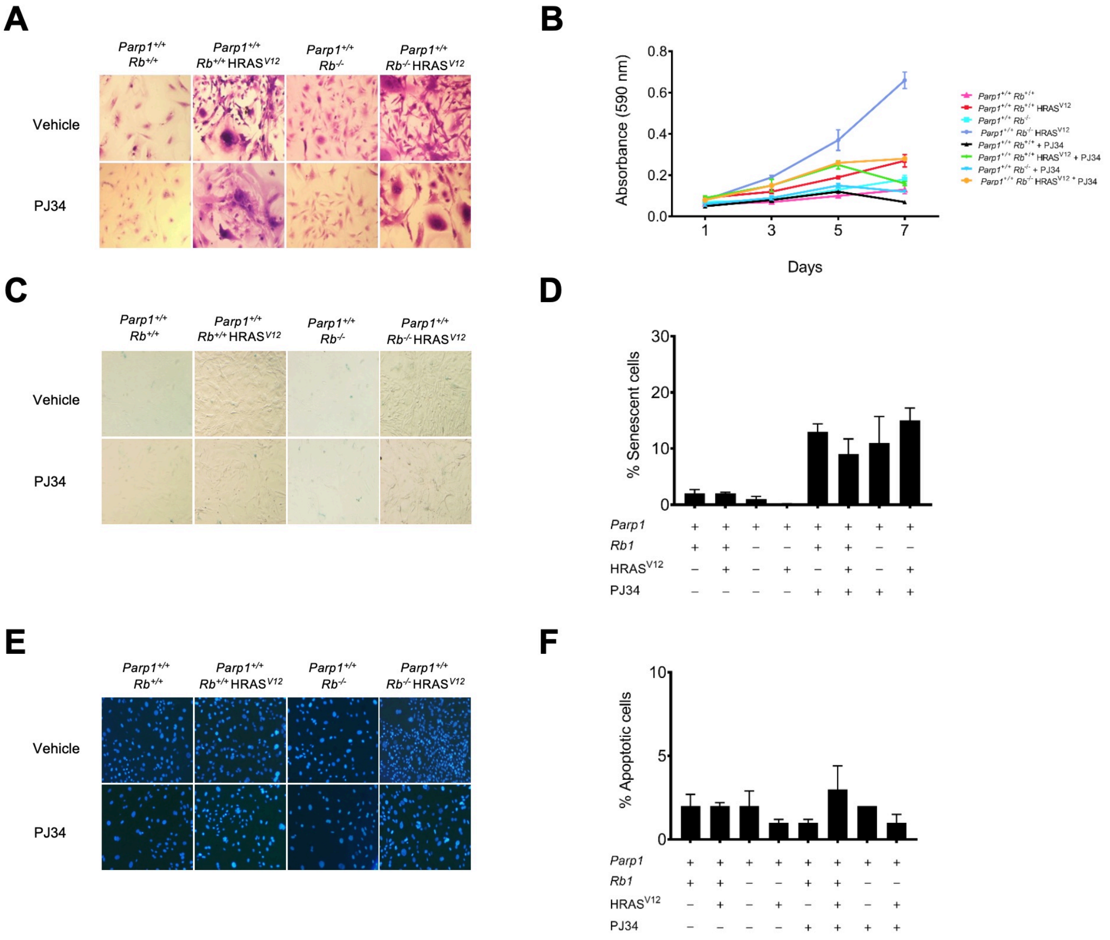

Supplementary Figure S2. A, morphology of *Parp1*$^{+/+}$ c*Rb*$^{flox/flox}$, *Parp1*$^{+/+}$ c*Rb*$^{flox/flox}$ *HRas*^{V12}, *Parp1*$^{+/+}$ c*Rb*$^{-/-}$ and *Parp1*$^{+/+}$ c*Rb*$^{-/-}$ *HRas*^{V12} astrocytes treated with vehicle or PJ34. B, proliferation curve of *Parp1*$^{+/+}$ c*Rb*$^{flox/flox}$, *Parp1*$^{+/+}$ c*Rb*$^{flox/flox}$ *HRas*^{V12}, *Parp1*$^{+/+}$ c*Rb*$^{-/-}$ and *Parp1*$^{+/+}$ c*Rb*$^{-/-}$ *HRas*^{V12} astrocytes treated with vehicle or PJ34. C, senescence assay by quantification of SA-β-galactosidase activity and representative photographs of the experimental groups. D, quantification of SA-β-galactosidase activity. Each experiment was repeated at least three times. E, apoptosis assay by staining of apoptotic nuclei (Hoechst 33258) and representative microphotographs of the experimental groups. F, quantification of apoptotic nuclei. Each experiment was repeated at least three times.

Effect of inhibition of PARP1 on primary astrocytes. Postnatal-day-3 astrocytes obtained from *Parp1*$^{-/-}$ c*Rb*$^{flox/flox}$, *Parp1*$^{-/-}$ c*Rb*$^{flox/flox}$ *HRas*^{V12}, *Parp1*$^{-/-}$ c*Rb*$^{-/-}$, or *Parp1*$^{-/-}$ c*Rb*$^{-/-}$ *HRas*^{V12} mice were treated with PJ34 (10 μM). A, morphological changes in cells stained with crystal violet. B, proliferation rate. Cells were stained with crystal violet, and cell number was determined by spectrophotometry. C,

percent of senescent cells, obtained by quantification of SA-β-galactosidase activity. D, representative images or of results presented in panel C. D, E, percent of apoptotics cells. Apoptotic nuclei were stained with Hoechst 33258. F, representative images of results presented in panel E.
